# Case Report: Minipuberty hormonal profile in *PPP1R12A*-related persistent Müllerian duct syndrome

**DOI:** 10.3389/fendo.2026.1882699

**Published:** 2026-09-15

**Authors:** Marie Voide, Federico Santoni, Lucia Bartoloni, Jenny Meylan-Merlini, Oliver Sanchez, Michael Hauschild, Nelly Pitteloud, Kanetee Busiah

**Affiliations:** 1 Faculty of Biology and Medicine, University of Lausanne, Lausanne, Switzerland; 2 Service of Endocrinology, Diabetology, and Metabolism, Lausanne University Hospital, Lausanne, Switzerland; 3 Service of Pediatric Surgery, Department of Mother-Woman-Child, Lausanne University hospital, Lausanne, Switzerland; 4 Pediatric Endocrinology, Diabetology, and Obesity Unit, Service of Pediatric, Department of Mother-Woman-Child, Lausanne University Hospital, Lausanne, Switzerland

**Keywords:** 46,XY disorder of sex development, *de novo* frameshift variant, minipuberty, persistent Müllerian duct syndrome, *PPP1R12A*-related urogenital and/or brain malformation syndrome

## Abstract

Persistent Müllerian duct syndrome (PMDS) is a rare difference/disorder of sex development and maturation characterized by the retention of Müllerian structures during fetal development in individuals with a 46,XY karyotype. It is typically due to mutations in anti-Müllerian hormone (*AMH*) or its receptor, anti-Müllerian hormone receptor type 2 (*AMHR2*). Here, we report a novel *de novo* heterozygous loss-of-function frameshift variant in protein phosphatase 1 regulatory subunit 12A (*PPP1R12A*; p.Lys197IlefsTer13) identified in a 46,XY neonate presenting with bilateral cryptorchidism and a Müllerian remnant detected on pelvic ultrasound. Hormonal evaluation during the first days of life and minipuberty revealed the expected activation of the hypothalamic-pituitary-gonadal axis and preserved gonadal function: serum inhibin B, 108 pg/mL; AMH, 174.4 pmol/L; and testosterone, 1.4 nmol/L at birth; and inhibin B, 259 pg/mL; AMH, 342 pmol/L; testosterone, 9.2 nmol/L; LH, 19.1 IU/L; and FSH, 10.4 IU/L, respectively. At 10 months, AMH (149.8 pmol/L) and inhibin B (76 pg/mL) concentrations were below the reported age-specific reference intervals, raising the possibility of evolving Sertoli cell dysfunction. *In silico* analyses predict a truncated protein and/or nonsense-mediated mRNA decay. The endocrine profile supports the hypothesis that the *PPP1R12A* variant may contribute to PMDS through abnormal duct development rather than primary gonadal failure. These findings broaden the endocrine phenotype associated with this syndrome and suggest a role for *PPP1R12A* in Müllerian duct regression and differentiation rather than in gonadal development.

## 1 Introduction

Persistent Müllerian duct syndrome (PMDS) (Online Mendelian Inheritance in Man (OMIM): 261550) is a rare difference/disorder of sex development. It is characterized by the incomplete regression of the Müllerian ducts during fetal development, leading to the presence of a uterus and fallopian tubes in otherwise fully masculinized 46,XY individuals (1). PMDS is most commonly caused by pathogenic variants affecting the anti-Müllerian hormone (AMH) signaling pathway, particularly in AMH or AMH receptor type 2 (AMHR2). However, in approximately 12% of PMDS cases, a link with *AMH*/*AMHR2* mutations cannot be established (2). Recent evidence suggests that mutations in the protein phosphatase 1 regulatory subunit 12A (PPP1R12A) gene are also associated with PMDS, often in association with urogenital and/or brain malformations, collectively referred to as *PPP1R12A*-related urogenital and/or brain malformation syndrome (UBMS) (OMIM: 602021) (1–6).

*PPP1R12A* is located at 12q21.2–q21.31 and encodes protein phosphatase 1 regulatory subunit 12A (also known as MYPT1). This subunit regulates protein phosphatase 1 (PP1) activity and substrate specificity, and it is involved in modulating cell motility, muscle contraction, and intracellular signaling. PPP1R12A plays a key role in smooth muscle relaxation, cell migration, cytoskeletal dynamics, and cancer progression, where its dysregulation can contribute to abnormal cell proliferation and metastasis. Experimental studies suggest that *PPP1R12A* participates in embryonic morphogenesis and mesenchymal remodeling, which are potentially relevant to Müllerian duct regression (3).

Minipuberty is an early postnatal and transient activation of the hypothalamic-pituitary-gonadal hormone axis, a critical phase in infant sexual development. Minipuberty appears to be essential for priming testicular function and is marked by a surge in gonadotropins such as follicle-stimulating hormone (FSH) and luteinizing hormone (LH), as well as testosterone, AMH, and inhibin B. In boys, minipuberty promotes masculinization, including testicular and penile growth, as well as further testicular descent (7).

The main objective of this report is to describe the neonatal and minipuberty profile in a male neonate with PMDS and a likely pathogenic variant in the *PPP1R12A* gene.

## 2 Case presentation

The patient was born at 39 weeks of gestation to nonconsanguineous parents of Moroccan and Kurdish origin. The patient had a family history of cryptorchidism on both the maternal and paternal sides of the family, although no detailed information could be obtained regarding these cases. There was no family history of fertility disorders.

At birth, the patient was small for gestational age (SGA), with a birth weight of 2,790 g (− 1.3 standard deviations [SD]), a height of 47 cm (−2 SD), and a head circumference of 35 cm (+ 0.2 SD). A clinical examination at birth revealed a genital tubercle measuring 2.5 cm × 0.9 cm (< P3), with an apical urethral meatal opening, incomplete foreskin, and a deviation of the median raphe. The labioscrotal folds were wrinkled and fused. The gonads were not palpable or descended, with no inguinal hernia. The external genital score (EGS) was 7.5 (8). There were no visible anal malformations. Otherwise, the neonatal examination revealed a simple sacral dimple with no physical asymmetry.

The patient experienced neonatal hypoglycemia (1.6 mmol/L) associated with SGA and neonatal polyglobulia. The hypoglycemia was treated with a 10% glucose infusion for 24 h, and the polyglobulia was managed with erythrocytapheresis. The patient presented with tachypnea, prompting a chest X-ray that revealed bilateral interstitial syndrome, which was treated with CPAP for 22 h. The infant exhibited jaundice with elevated total (346 μmol/L) and direct bilirubin (12 μmol/L) levels (normal < 10 μmol/L), which was treated with phototherapy for 2 days. The transfontanellar ultrasound was normal.

In the context of neonatal impalpable gonads and incomplete foreskin, we conducted a complete biological, radiological, and genetic investigation of differences/disorders of sex development (DSD). The karyotype was 46,XY. The patient was male-assigned. Abdominal ultrasound on day 5 revealed a persistent Müllerian duct (2.5 cm × 1 cm) with fluid in the cervix and no visible gonads. The adrenal glands were in a normal position and had normal morphology. No evidence of a low fixed medulla was found.

Biological assessments were conducted at birth and at 1.5, 2.5, 6, and 10 months (**Table 1**). LH, FSH, and AMH were assayed using the Roche Elecsys electrochemiluminescent immunoassay (ECLIA) (Roche™ Diagnostics, Basel Switzerland), and inhibin B was assayed using the Gen II ELISA (Beckman Coulter, California, USA). Reference values for inhibin B were derived from studies employing the same analytical methodology used in our laboratory (7, 9).

**Table 1:** Patient longitudinal hormonal profile.

Age	34 h	4 days	1.5 months	2.5 months	6 months	10 months
FSH (IU/L)	< 0.3	–	10.4	9.8	8.3	3.7
LH (IU/L)	< 0.3	–	19.1	13.0	1.5	0.5
Inhibin B (pg/mL) (reference values) (7, 9)	108 (90–310)	116 (90–310)	259 (105–444)	–	–	76 (122–589)
AMH (pmol/L) (reference values)	174.4 (78–606)	194.7 (159–1,186)	342.1 (235–1,126)	–	–	149.8 (235–1,126)
Testosterone (nmol/L) LC-MS/MS	1.4	0.3	9.2	7.5	< 0.1	< 0.1

Reference values for AMH and inhibin B were derived from studies employing the same analytical methodology used in our laboratory (7, 11). The reference range in men is the 2.5th–97.5th percentile reference interval (10). FSH: 1–30 days, 0.28–6.33; 31–60 days, 0.67–6.30; 61–120 days, 0.57–5.81; 121–365 days, 0.19–3.63 (limit of quantification = 0.3 UI/L). LH: 1–30 days, 0.72–13.53; 31–60 days, 1.29–9.16; 61–120 days, 0.87–9.59; 121–365 days, 0.10–8.06 (limit of quantification = 0.3 UI/L). Testosterone: 1–30 days, 1.37–13.00; 31–60 days, 2.18–13.25; 61–120 days, 2.31–12.2; 121–365 days, 0.42–5.77 (limit of quantification = 0.1 nmol/L). AMH (limit of quantification = 0.1 pmol/L); inhibine B (limit of quantification = 15 pg/mL).

*FSH*, follicle-stimulating hormone; *LH*, luteinizing hormone; *AMH*, anti-Müllerian hormone.

Minipuberty assessments revealed slightly elevated FSH (10.4 IU/L) and LH (19.1 IU/L) concentrations, with detectable testosterone levels (9.2 nmol/L), while AMH (342 pmol/L) and inhibin B (259 pg/mL) were within normal reference values (reference intervals are provided in **Table 1**). The FSH and LH concentrations were 1.6- and 2.0-fold above the age-specific upper reference limits, respectively (10).

At 10 months, a comprehensive clinical and biological evaluation revealed a reduced anogenital distance ratio (AGDl/u) of 0.38, with AGDl measuring 2.5 cm and AGDu 6.5 cm, in accordance with established references (9). This low AGDl/u ratio may reflect impaired fetal androgen action despite detectable postnatal testicular function. The EGS remained 7.5 (8). The patient presented with thick eyebrows and hypotelorism, which subsequently resolved with growth. We observed decreased AMH (from 194.7 pmol/L at 4 days to 149.8 at 10 months) and inhibin B (from 116 to 76 pg/mL) concentrations, compatible with emerging Sertoli cell dysfunction (reference intervals are provided in **Table 1**). Inhibin B and AMH concentrations were 1.6- and 1.5-fold below the age-specific lower reference limits, respectively (7, 9).

At 3 years of age, a laparoscopy revealed Müllerian derivatives extending bilaterally toward the intraabdominal gonads. The gonads appeared to be testes with no visible follicles and a homogeneous white capsule. The right and left vessels were orthotopic.

A staged surgical strategy was planned to achieve bilateral orchiopexy and incision of the Müllerian derivatives while preserving the gonadal vascular supply and the vas deferens.

Exome sequencing revealed a heterozygous loss-of-function frameshift variant NM_002480.3:c.589_590insT (p.Lys197IlefsTer13) in the *PPP1R12A* gene. The presence of the variant was confirmed by PCR. This variant was absent in both parents (*de novo*) and is absent from the Genome Aggregation Database (gnomAD), as well as from clinically relevant databases such as ClinVar and LOVD. The variant is located in exon 4 (out of 25). It is a single-nucleotide insertion causing a frameshift that introduces a premature stop codon after 13 codons. According to Auto Pathogenic very strong (PVS1) (PMID: 32442321), this variant leads to nonsense-mediated mRNA decay (NMD), with loss of transcripts and, consequently, loss of protein function. Based on this evidence, the variant was classified as likely pathogenic according to the ACMG classification (PVS1 Strong, PM2, PM6). No additional reportable pathogenic (P), likely pathogenic (LP), or clinically relevant uncertain variants were identified in the analyzed DSD-associated genes, and no clinically relevant copy-number variants were detected using ExomeDepth. The average coverage was 100 ×, and all genes were covered at > 30× for 95% of their exonic length. The presence of CNVs was also investigated using ExomeDepth, and no clinically relevant CNVs were identified. The VarSome Clinical package was used for the analysis (**Figure 1**).

**Figure 1:**
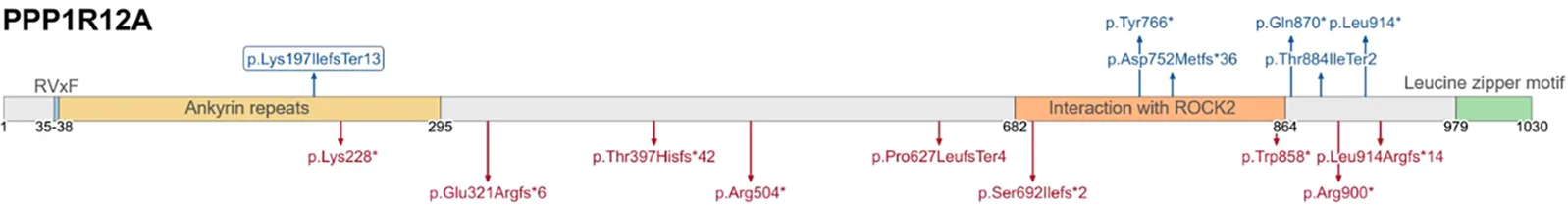
Localization of the DSD variant on the myosin phosphatase regulatory subunit encoded by PPP1R12A (Original Figure created based on data from PPP1R12A - Protein phosphatase 1 regulatory subunit 12A - Homo sapiens (Human) | Variants viewer | UniProt (https://www.uniprot.org/uniprotkb/O14974/variant-viewer) and Tong et al (2024) (11)) Shown here is the RVKF motif in blue, which is important for interaction with PPP1CB, and the interaction domain with Rho-associated coiled-coil-containing protein kinase 2 (ROCK2) in orange. Mild variants (in blue) are associated with less severe external genitalia phenotypes, such as small genital tubercle. Severe variants (in red) are associated with more pronounced undervirilization of the external genitalia, including notably hypospadias or posterior labial fusion. The outlined rectangles indicates the variant of interest of this publication. In the study by Hughes et al (2020)(3), another variant located in a non-coding region (c.793-1G>A) is reported, whereas Picard et al (2022)(1) described a different variant involving complex rearrangements.

Pathogenic loss-of-function variants in *PPP1R12A*, a gene intolerant to LoF (haploinsufficient with pLI = 1, LOEUF = 0.2), are associated with the autosomal dominant *PPP1R12A*-related UBMS. Therefore, further investigations for neurological and urogenital anomalies were recommended. At age 3, the neurological examination was normal. Transfontanellar ultrasound and spinal cord imaging were unremarkable. The family declined further neurological imaging. The identified genital anomalies were consistent with persistent Müllerian derivatives. Abdominal ultrasonography and laparoscopy revealed no additional malformations.

The patient presented here aligns with the phenotypic and genotypic spectrum of the 16 previously published 46,XY cases of *PPP1R12A*-related UBMS. **Figure 1** (11) and **Table 2** summarize all variants and phenotypes reported to date in association with this syndrome (1, 3, 5, 6).

**Table 2:** Review of reported cases of *PPP1R12A*-related UBMS.

Case (reference)	Variant	External genitalia	Internal genitalia	Hormonal profile	Neurological	Others
1 (3)	c.793-1G>A	Not described	Not described	Not evaluated	Agenesis of the corpus callosum, ADHD	Renal asymmetry, kyphoscoliosis, stiff joints, decreased subcutaneous fat
2 (3)	c.2739_2740delCT (p.Leu914ArgfsTer14)	Small tubercle genital, chordee, scrotal hypospadias	Bilateral ectopic gonads, Müllerian derivatives	Not evaluated	Head CT unremarkable	
3 (3)	c.1510C>T (p.Arg504Ter)	Glandular hypospadias, chordee	Not described	Not evaluated	Dysgenesis of the corpus callosum, absent septum pelludicidum, Chiari malformation, cortical dysplasia/polymicrogyria,grey matter heterotopia, developmental delay	
4 (3)	c.2573G>A (p.Trp858Ter)	Hypospadias	Ectopic gonads (ovaries), Müllerian derivatives	Not evaluated	Leukomalacia, developmental delay, bilateral rod and cone dysfunction, decreased vision, latent nystagmus	
5 (3)	c.2073dupA (p.Ser692IlefsTer2)	Female phenotype	Müllerian derivatives, streak gonads	Not evaluated	Not evaluated	
6 (3)	c.2698C>T (p.Arg900Ter)	Small genital tubercle, posterior fusion of labia majora	Urogenital sinus	Not evaluated	Not evaluated	
7 (3)	c.960dupA (p.Glu321ArgfsTer6)	Penile hypospadias	Bilateral ectopic gonads, Müllerian derivatives	Not evaluated	Brain MRI unremarkable	
8 (3)	c.1189delA (p.Thr397HisfsTer42)	Female phenotype	Streak gonads, Müllerian derivatives	Not evaluated	Brain MRI unremarkable	
9 (3)	c.681dupT (p.Lys228Ter)	Small tubercle genital, posterior labial fusion, labial rugation and pigmentation	Müllerian derivative, ovaries	Not evaluated	Not evaluated	
10 (6)	c.1880delC (p.Pro627LeufsTer4)	Small tubercle genital, majora and minora labia	Ectopic gonad (ovotestes), no müllerian derivatives	3months : androstenedione 0.42 ng/mL (0.3-3.3), dehydroepiandrosterone sulfate 2.54 mcg/dL (3-124), testosterone 148.17 ng/dL (0.58-33), 17α-hydroxy-progesterone 2.82 ng/mL	Pseudocyst in the temporal region	Left inguinal herniation, multiple superficial infantile hemangiomas
11 (5)	c.2666+3A>Go. (p-Thr884IleTer2)	Small genital tubercle, hypospadias, dark testicular skin	Gonads (testicles)	Not evaluated	No abnormalities (IRM)	
12 (1)	c.2253delA (p.Asp752MetfsTer36)	Fully virilized OGE	Bilateral ectopic gonads (normal testicular tissue), Müllerian derivatives	AMH, testosterone, LH and FSH normal for age	Not described	Proximal jejunal atresia
13 (1)	c.2741T>A (p.Leu914Ter)	External male phenotype	Bilateral ectopic gonads, Müllerian derivatives	3days : AMH 201pmol/l, Testosterone 45pg/L	Not described	Jejunal atresia type IIIb
14 (1)	c.2298C>A (p.Tyr766Ter)	External male phenotype	Gonads ectopics	Not evaluated	Small cerebellum	Small intestinal atresia
15 (1)	Complex rearrangement	External male phenotype	Ectopic gonads, Müllerian derivatives	22years:FSH 13.1 U/ml, LH 10.9 U/ml, testosterone 21.8 nmol/l 22,5years :FSH 126.4 mUI, LH 10.9 mUI, testosterone 7.9 nmol/l, AMH 10.1 pmol/l	Not described	Partial esophageal atresia
16 (1)	c.2608C>T (p.Gln870Ter)	Not described	Bilateral ectopic gonads, Müllerian derivatives	7years : AMH 171.36 pmol/l 18years FSH 16.4 mUI, Testosterone 22.88 nmol/	Not described	
17	c.589_590insT (p.Lys197IlefsTer13)	Small genital tubercle, incomplete foreskin, deviation raphe median, fused and wrinkle labioscrotal folds	Bilateral ectopic gonads, Müllerian derivative	Cf. **Table 1**	Not observed	Mild dysmorphia

## 3 Discussion

We report a newborn from a nonconsanguineous couple, presenting with an attenuated phenotype of *PPP1R12A*-related UBMS. While *PPP1R12A*-related UBMS is typically associated with both urogenital anomalies (including persistent Müllerian remnants) and brain malformations, our patient exhibited isolated urogenital features without detectable neurological involvement, as confirmed by prenatal ultrasonography and postnatal transfontanellar ultrasound.

The detailed longitudinal hormonal profile at birth, minipuberty, and at 10 months of life is consistent with a previously undescribed pattern of possible progressive testicular dysfunction in *PPP1R12A*-related UBMS. Hormonal evaluations during the neonatal period and minipuberty assessments revealed normal AMH and inhibin B concentrations, consistent with preserved Sertoli cell function. Detectable testosterone concentrations at both day two of life and during minipuberty further support preserved Leydig cell function and the absence of defects in testosterone biosynthesis. Altogether, these findings argue against the presence of a major prenatal primary congenital gonadal dysgenesis that would be expected to result in the persistence of Müllerian structures. The elevated LH concentration (2.0-fold above the age-specific upper reference limit) (10) in the presence of an age-appropriate testosterone concentration may be compatible with compensated Leydig cell dysfunction; however, physiological variability during minipuberty must be considered. We also observed a decline in AMH and inhibin B concentrations after minipuberty (1.5- and 1.6-fold below the age-specific lower reference limits; reference intervals are provided in **Table 1**) (7, 9). Altogether, these findings suggest a possible progressive gonadal insufficiency that might be secondary to the thermal effects of the intraabdominal location of the gonads, although progressive gonadal dysgenesis cannot be excluded. Indeed, elevated intraabdominal temperature is deleterious to the gonads. Prolonged intraabdominal cryptorchidism has been associated with progressive testicular impairment, and delayed diagnosis and management may contribute to cumulative gonadal damage. In our case, the decreased serum concentrations of AMH and inhibin B suggest impaired Sertoli cell function, which may reflect the cumulative impact of 10 months of bilateral intraabdominal cryptorchidism (12–14) (**Table 1**).

As only an early hormonal assessment is available, the clinical significance of the observed findings remains uncertain. While they may indicate progressive gonadal dysgenesis or reflect the impact of prolonged bilateral intraabdominal cryptorchidism, physiological fluctuations and analytical variability should also be considered. Long-term follow-up, including repeated assessments of Sertoli and Leydig cell markers and evaluation during puberty, will be essential to determine the trajectory of gonadal function and future reproductive potential. Furthermore, this case report is limited by the absence of functional studies and histological data, which preclude a more comprehensive characterization of the underlying gonadal pathology.

This case is consistent with the involvement of genes other than *AMH* and *AMHR2* in the persistence of Müllerian derivatives. Our patient’s hormonal profile, characterized by normal AMH and testosterone levels during minipuberty, supports the hypothesis that *PPP1R12A*-related UBMS may be included within the PMDS spectrum, as suggested by Picard et al. (1), who classified their cases as PMDS based on persistently normal AMH and testosterone levels during childhood and adulthood (1).

The absence of clinically apparent neurological malformations in our patient is consistent with the wide phenotypic variability reported in *PPP1R12A*-related UBMS. However, brain MRI was not performed, as the family declined further neuroimaging, and evaluation was limited to transfontanellar ultrasound. Therefore, subtle structural brain abnormalities cannot be definitively excluded. While some cases present with severe brain anomalies, such as holoprosencephaly or corpus callosum agenesis, others, like ours, exhibit isolated urogenital features. In a study by Hughes et al. (3), only two patients showed an association between brain and genitourinary anomalies, and three 46,XX individuals with neurological involvement did not have detailed genital documentation. This phenotypic spectrum underscores the need for comprehensive evaluation in all patients with *PPP1R12A* variants, regardless of the severity of their initial presentation.

Comparison with previously reported *PPP1R12A*-related UBMS cases revealed no clear correlation between variant location and phenotypic severity (**Figure 1**). Although *PPP1R12A* and its encoded protein, MYPT1, have been implicated in cell adhesion and migration processes, the pathophysiological mechanisms responsible for *PPP1R12A*-related UBMS remain unknown. In particular, the role of *PPP1R12A* in Müllerian duct regression and its potential interaction with the AMH signaling pathway have yet to be established (1, 3, 5). The novel hormonal findings of our case expand the known spectrum of *PPP1R12A*-related UBMS phenotypes and underscore the importance of systematic, early, and comprehensive hormonal assessment in infants with DSD, including bilateral cryptorchidism, regardless of the apparent severity. In our clinic, a standardized clinicobiological–radiological investigation protocol is implemented to ensure early diagnosis and appropriate management. Physicians should remain vigilant for any phenotype associated with bilateral cryptorchidism, such as micropenis or incomplete foreskin, as these may be the only external indicators of underlying PMDS or progressive gonadal dysgenesis.

## 4 Conclusion

In conclusion, this case of isolated bilateral cryptorchidism led to the identification of a novel *PPP1R12A* variant with persistent Müllerian structures and suggests evolving Sertoli cell dysfunction, highlighting the need for longitudinal endocrine follow-up. The temporal decline in Sertoli cell markers raises the possibility of postnatal testicular impairment, potentially related to prolonged bilateral intraabdominal cryptorchidism, although intrinsic gonadal involvement and physiological or analytical variability cannot be excluded. Longitudinal follow-up, particularly after orchiopexy, including serial assessment of Sertoli and Leydig cell function and evaluation through puberty, will be crucial to determine the reversibility of this impairment and its long-term implications for spermatogenesis.

## Data Availability

The original contributions presented in the study are included in the article/supplementary material. Further inquiries can be directed to the corresponding author.
